# 

**Published:** 1891-02

**Authors:** 


### CONTENTS.

### COMMUNICATIONS.

Artificial Tooth Crowns.......... 57
Five Cases of Removal of the Jaw for Tumor.......... 63
General Manner of Conducting Dental Societies.......... 70
Diseases of the Teeth and their Relation to the General Health .......... 72

### SELECTIONS.

Peroxide of Hydrogen and Ozone—Their Antiseptic Properties.......... 76
The Relation of Diet to Personal Beauty.......... 80
Ambidexterity .......... 82
Diet in Diphtheria with Special Consideration of the Proper Food for Children.......... 84
Feeding the Young.......... 87
Suggestions of a Method of Changing Bodily Form by the Administration of Certain Food.......... 89
The Ten Health Commandments.......... 91
A Case of occlusion of Steno's Ducts.......... 93
Selection from a Clinical Lecture.......... 94
Treatment of Diphtheria.......... 95
What is the Best Method for Preventing Infection of Operative Wounds?.......... 96
The Action of Chloroform and Ether.......... 96
Reunion of Cut-Off Tongue.......... 97
The Question of Shock during Operations under Anæsthetics.......... 98
Bone Grafting.......... 99
The Second Annual Meeting of the Dental Protective Association of the United States.......... 100

### EDITORIAL.

Chancres of the Fingers.......... 102
Instruments .......... 105
Cleanliness.......... 106
Acid Fruits.......... 108

